# CXCL5 Downregulation in Villous Tissue Is Correlated With Recurrent Spontaneous Abortion

**DOI:** 10.3389/fimmu.2021.717483

**Published:** 2021-09-17

**Authors:** Sainan Zhang, Jinli Ding, Jiayu Wang, Tailang Yin, Yan Zhang, Jing Yang

**Affiliations:** ^1^Reproductive Medical Center, Renmin Hospital of Wuhan University & Hubei Clinic Research Center for Assisted Reproductive Technology and Embryonic Development, Wuhan, China; ^2^Department of Clinical Laboratory, Renmin Hospital of Wuhan University, Wuhan, China

**Keywords:** CXCL5, recurrent spontaneous abortion, invasion, migration, trophoblast

## Abstract

Recurrent spontaneous abortion (RSA) affects 5% of childbearing-age women worldwide. Inadequate trophoblast invasion is one of the main reasons for the development of RSA; however, the underlying molecular mechanisms for RSA have not been fully understood, and further explanation is urgently needed. C-X-C motif chemokine ligand 5 (CXCL5) is reported to contribute to the invasion of cancer cells, and its aberrant expression is associated with the cellular process of tumor pathology. Considering the high behavioral similarity between trophoblast cells and cancer cells, we hypothesized that CXCL5 may influence trophoblast invasion, and its expression levels in villous tissue may be correlated with RSA. In this study, we firstly investigated the CXCL5 expression in placental villous tissues of 15 RSA patients and 13 control patients, and the results showed that CXCL5 levels were significantly lower in villous tissue from RSA patients than those of the controls. Further *in vitro* experiments presented that recombinant human CXCL5 can enhance trophoblast migration, invasion, and epithelial-to-mesenchymal transition (EMT) process. We also demonstrated that CXCL5 exerted these effects on trophoblast cells through PI3K/AKT/ERK1/2 signaling pathway. In conclusion, these data indicate that CXCL5 downregulation in human villous tissue is correlated with RSA. Additionally, we found that estrogen, progesterone, human chorionic gonadotropin, and decidual stromal cells can regulate CXCL5 and chemokine receptor 2 (CXCR2) expression of trophoblast in a cell manner.

## Introduction

Recurrent spontaneous abortion (RSA) is defined as two or more times of consecutive miscarriages before 20 weeks of gestation and impacts approximately 5% of childbearing-age women throughout the world (1–3). There is no unified theory in the pathogenesis of RSA. Several known factors for RSA include endocrine diseases, genetic abnormalities, immune diseases, and anatomical abnormalities, and approximately 50% of RSA cases are unexplained (4). The underlying molecular and cytological mechanisms for RSA also remain largely enigmatic. As the most common pregnancy-associated complication, RSA seriously disturbs the physical and mental health of the female population and is also frustrating for the physician. Trophoblast invasion is a key event during pregnancy and plays a vital role in the process of embryo implantation and placentation (5). In the early stage of pregnancy, trophoblast cells from the trophectoderm differentiate into two main lineages, villous cytotrophoblasts (VCTs) and extravillous trophoblasts (EVTs). Subsequently, EVTs undergo migration and invasion into the maternal decidua and myometrium, during which process embryos are anchored to the uterine wall and the uterine vessels are remodeled to form the low-resistance spiral arteries (6, 7). Proper trophoblast invasion is necessary for a successful pregnancy. Studies show that poor trophoblast invasion is related to a series of pregnancy complications, including RSA, preeclampsia, and fetal growth restriction (8, 9). Inadequate invasion of the trophoblast is even reported to be one of the main reasons for RSA (10–12). Therefore, exploring the factors that affect trophoblast invasion is of great significance for improving our understanding of the pathogenesis of RSA. Many molecules including hormones, chemokines, and growth factors, are associated with trophoblast invasion within the maternal–fetal microenvironment. Of these, chemokines are a large family of small-molecular-weight peptides that are initially involved in the pro-inflammatory process (13). Recently, studies have demonstrated that a wide range of chemokines are expressed at the maternal–fetal interface, and these chemokines can directly participate in the regulation of trophoblast invasion and the establishment of maternal–fetal tolerance (14, 15).

C-X-C motif chemokine ligand 5 (CXCL5), also known as epithelial neutrophil-activating peptide 78 (ENA-78), is a member of the CXC chemokine subfamily and is originally identified in neutrophils (16). It has been demonstrated that CXCL5 is a potent mediator of neoangiogenesis and is mainly expressed in epithelial cancer cells and immune cells (17). Chorionic trophoblast and amniotic epithelium membranes have also been confirmed to express CXCL5 (18). Abnormal expression of CXCL5 is found to be correlated with a large number of diseases, such as autoinflammation, cancer, obesity, and diabetes (19–21). CXCL5 exerts its action by binding to its G-protein-coupled receptors chemokine receptor 2 (CXCR2), and the CXCL5/CXCR2 axis has been widely investigated in various types of cancers. Numerous studies have confirmed that CXCL5 can induce the epithelial-to-mesenchymal transition (EMT) process in cancer cells and thus promotes cancer cells invasion and metastasis (22, 23). Trophoblast cells and cancer cells share striking behavioral similarities in invasion, migration, and proliferation capacities (24). Whether CXCL5 can affect trophoblast invasion has not been reported. A previous study identified that human villi expressed CXCR2 (25). It was also found that as one of the ligands of CXCR2, IL-8, was expressed in human decidua and trophoblast and promoted trophoblast migration and invasion in an autocrine or paracrine manner (26). Similar results were found in CXCL3 (27). These findings draw our attention to the CXCL5. Additionally, one study evaluated the relationship between the levels of chemokine and the risk of miscarriage. The study found that elevated CXCL5 levels from serum samples were associated with an increased risk of miscarriage as the collection-outcome interval increased, although the authors did not observe statistical significance (28). Based on these results, we hypothesized that CXCL5 may exert an effect on trophoblast migration and invasion and thus could participate in the occurrence of RSA.

In the present study, we compared CXCL5 levels in the placental villous tissue of RSA patients and control patients. We also investigated its effects on the trophoblast and explored the underlying mechanism. Our results showed that the expression of CXCL5 was significantly lower in RSA patients. We also further demonstrated that CXCL5 can induce the EMT process through PI3K/AKT/ERK1/2 signaling pathway and thus promoted trophoblast invasion and migration. Collectively, these data provide the first evidence that CXCL5 downregulation in villous tissue is correlated with RSA. In addition, we found that local factors, including estrogen (E), progesterone (P), human chorionic gonadotropin (HCG), and decidual stromal cells (DSCs) regulated CXCL5 and CXCR2 expression of trophoblast cells.

## Materials and Methods

### Patients and Clinical Samples

This study was approved by the ethics committee of Renmin Hospital of Wuhan University, and consent was obtained from each patient before sample collection. Fifteen patients with RSA and 13 control patients from the reproductive medical center in Renmin Hospital of Wuhan University were included between December 2017 and October 2019. Clinical data and placental villous tissue samples were obtained from the two groups. RSA was defined as the loss of two or more sequential pregnancies with the same partner before a gestational of 20 weeks. The exclusion criteria were as follows: a) symptoms of endocrine or metabolic diseases, such as hyperthyroidism and diabetes; b) karyotype abnormality; c) infection based on routine leucorrhea examination; and d) uterine abnormality. Women who had healthy pregnancies and underwent selective pregnancy terminations for non-medical reasons constituted the control group. The villous tissues were collected immediately following surgery: one portion was fixed with 4% paraformaldehyde for paraffin embedding in blocks, and the others portion was stored in liquid nitrogen.

### Immunohistochemistry

The paraffin-embedded villous tissues were cut into 4-μm-thick sections and dehydrated in a graded series of ethanol. Endogenous peroxidase activity was blocked with 3% H_2_O_2_, and non-specific binding was blocked with 5% bovine serum albumin (BSA) for 15 min. Next, the samples were incubated at 37°C with primary rabbit anti-human CXCL5 antibody (1:500; Affinity, Cat: DF9919), anti-E-cadherin (1:500; ProteinTech; Cat: 20874-1-AP) and anti-N-cadherin (1:100; ProteinTech; Cat: 22018-1-AP) antibodies. All sections were washed three times with PBS and then incubated with secondary antibodies. The reaction was detected with 3,3′-diaminobenzidine (DAB), and the sections were counterstained with hematoxylin. Five visual fields were selected, and the staining was observed under an Olympus BX51+DP70 microscope at ×200 and ×400 magnification. The images were analyzed with ImageJ (1.52a, National Institutes of Health, USA).

### Immunofluorescence

The paraffin-embedded human villous tissues were cut into 2-μm-thick sections. Deparaffinization, hydration, and antigen retrieval of the sections were carried out under proper conditions. The samples were incubated with rabbit anti-human cytokeratin 7 (CK7) (1:100; ProteinTech, Cat: 15539-1-AP) and rabbit anti-human CXCR2 (1:50; ProteinTech, Cat: 20634-1-AP) primary antibodies. After that, the samples were incubated with fluorescence-labeled secondary antibody for 1 h and counterstained with 4′-6-diamidino-2-phenylindole (DAPI) (Beyotime, Shanghai, China). A confocal laser scanning microscope (Olympus FV1000, Japan) was used to observe the fluorescence signal. Five visual fields with tissue were selected for analysis. The pixel intensity per unit area was assessed using ImageJ (1.52a, National Institutes of Health, USA).

### Cell Culture, Reagents, and Treatments

HTR-8/SVeo cell line was obtained from the China Center for Type Culture Collection (Wuhan, China) and cultured in DMEM-F12 medium (Gibco, USA) supplemented with 10% fetal bovine serum (FBS) (Gibco, USA). Human endometrial stromal cells were purchased from the BeNa culture collection and induced toward DSCs according to a previous method, with some modifications (29, 30). HTR-8 cells were seeded on a 6-well plate (2 × 10^5^ cells/well) and placed in an incubator with 5% CO_2_ at 37°C. A co-culture model of HTR-8 cells and DSCs was established *via* a Transwell co-culture system (0.4-μm pore size, Corning, USA). In brief, HTR-8 cells were seeded into the lower chambers, and DSCs were placed into the upper chambers at different ratios (DSCs: HTR-8 cells 1:4; 1:1; 2:1) for 48 h before harvest.

Recombinant human CXCL5 (Absin, Shanghai, China) was used at the concentration of 50 and 100 ng/ml according to the manufacturer’s instruction. LY294002 (PI3K/AKT inhibitors) and PD98059 (ERK1/2 inhibitors) were purchased from MedChemExpress, China, and used at concentrations of 20 and 30 μM, respectively. Hormone concentrations used in the current experiment were E (10^−7^ M), P (10^−8^ M), and HCG (5 kU/L).

### Quantitative Real-Time PCR

Total RNA was extracted from cells and tissues using TRIzol reagent (Invitrogen, USA) according to the manufacturer’s instructions. Reverse transcription was conducted with the PrimeScript RT reagent kit (Takara, Japan). RT-PCR was performed with a SYBR Premix Ex Taq II kit (Takara, Japan) on a 7500 detection system (Applied Biosystems, Foster City, CA, USA). 2^−ΔΔ Ct^ method was determined to calculate and quantify the gene expression. Primers were designed with computer assistance based on gene sequences available in GenBank, and the sequences of primers are listed in **Table 1**.

**Table 1:** Primer sequences.

Primers		Sequences (5′–3′)
GAPDH	F	CACTGGGCTACACTGAGCAC
	R	AGTGGTCGTTGAGGGCAAT
CXCL5	F	AGCTGCGTTGCGTTTGTTTAC
	R	TGGCGAACACTTGCAGATTAC
CXCR2	F	CCTGTCTTACTTTTCCGAAGGAC
	R	TTGCTGTATTGTTGCCCATGT

GAPDH, glyceraldehyde 3‐phosphate dehydrogenase; CXCL5, C-X-C motif chemokine ligand 5; CXCR2, chemokine receptor 2.

### Western Blotting

Cells were harvested and lysed with radioimmunoprecipitation assay (RIPA) lysis buffer, and the lysates were centrifuged at 4°C for 15 min to collect the supernatant. A bicinchoninic acid (BCA) assay kit (Beyotime, Shanghai, China) was used to measure protein concentrations. After boiling with a 5× loading buffer (Beyotime, Shanghai, China) at 95°C for 5 min, 40 μg of protein of each sample was electrophoresed *via* 10% sodium dodecyl sulfate–polyacrylamide gel and transferred to polyvinylidene difluoride (PVDF) membranes (Millipore) for blocking 1 h at room temperature with 5% BSA. The following primary antibodies were incubated together with the membranes overnight at 4°C: rabbit anti-E-cadherin (dilution 1:1,000; ProteinTech; Cat: 20874-1-AP), anti-N-cadherin (dilution 1:1,000; ProteinTech; Cat: 22018-1-AP), anti-vimentin (dilution 1:1,000; ProteinTech; Cat: 10366-1-AP), anti-GAPDH (dilution 1:1,000; ProteinTech; Cat: 10494-1-AP), and anti-tubulin (dilution 1:1,000; ProteinTech; Cat: 10094-1-AP). The secondary antibodies were incubated for 1 h at room temperature the next day. Finally, the protein bands were visualized with an enhanced chemiluminescence (ECL) detection system (Bio-Rad, Hercules, CA, USA), and the relative band intensities were calculated with ImageJ (1.52a, National Institutes of Health, USA).

### Invasion Assay

A 24-well plate Transwell insert (8-µm pore size, Corning, USA) coated with Matrigel matrix (Corning, USA) was used to detect the invasion ability of the cell. In brief, HTR-8 cells (2 × 10^4^) were seeded in the upper chamber of each insert in a 200 µl FBS-free DMEM-F12 medium. The lower chamber was filled with 500 µl of DMEM-F12 medium containing 10% FBS. The plate was placed in an incubator with 5% CO_2_ at 37°C for 48 h. Afterward, cells that had invaded the lower chamber were fixed with 4% paraformaldehyde, stained with 0.5% crystal violet, and quantified. The average cells number from five fields at a magnification of ×200 was reported.

### Scratch Wound Healing Assay

Cell migration ability was evaluated with scratch wound healing assay. When cells reached 80%–90% confluence, a scratch wound was made on the monolayer of cells with a 200-µl pipette tip and gently washed three times with PBS before the serum-free medium was added. The 6-well plate was incubated with 5% CO_2_ at 37°C for 48 h. Pictures of the wound were taken at 0 and 48 h. The wound area was calculated using ImageJ.

### Statistical Analysis

Quantitative data were expressed as the mean ± standard deviation (SD) and analyzed by independent t-test. Categorical data were compared by the Mann–Whitney U-test. All experiments were independently repeated at least three times. Figures were performed by GraphPad Prism version 6.0 (GraphPad Software, San Diego, CA). All *p*-values were two-sided and statistical significance was established as *p* < 0.05. All analyses were conducted using SPSS 22.0 (IBM SPSS, USA).

## Results

### Clinical Baseline Characteristics

Collected data at baselines included maternal age, gestational week, body mass index, number of pregnancies, number of live births, and number of miscarriages. Detailed information is listed in **Table 2**. No differences were found between the RSA group and the control group in terms of age, gestational week, body mass index, and the number of pregnancies. In addition, the RSA group showed a higher number of miscarriages and a significantly lower number of live births than the control group (*p* < 0.01).

**Table 2:** Clinical characteristics of the population.

Primers	Control (n = 13)	RSA (n = 15)
Age (years)	30.17 ± 2.05	30.63 ± 3.97
BMI (kg/m^2^)	21.17 ± 4.15	22.74 ± 3.89
Gestation week	7.17 ± 2.15	7.67 ± 2.01
Number of pregnancy	1.47 ± 0.35	2.59 ± 0.82
Number of miscarriage	0.77 ± 0.35	2.67 ± 0.55**
Number of live birth	1.56 ± 0.35	0.00 ± 0.00**

RSA, recurrent spontaneous abortion; BMI, body mass index.

**p < 0.01.

### Expression of CXCL5 Is Downregulated in Villous Tissues of Recurrent Spontaneous Abortion Patients

**Figure 1:**
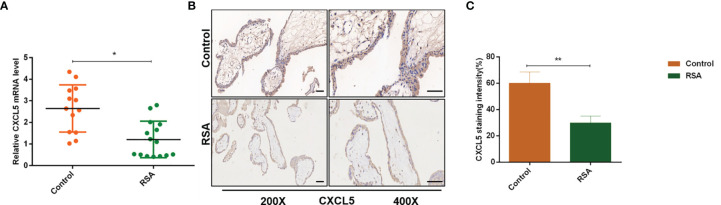
CXCL5 expression in human placental villous tissue from the RSA group and the control group. **(A)** Expression of CXCL5 mRNA in the RSA group (n = 15) and control group (n = 13) by RT-PCR. **(B, C)** Representative immunohistochemical staining images and quantification of CXCL5 in the RSA group (n = 15) and normal group (n = 13). Relative gene expression was normalized to GAPDH. ImageJ is used to quantify the staining intensity. Magnification, ×200 and ×400. Scale bars = 100 μm. Data are presented as the mean ± SD. Results are reported as fold change compared with the control group. (*p < 0.05, **p < 0.01). RSA, recurrent spontaneous abortion; mRNA, messenger RNA; RT‐PCR, quantitative real-time polymerase chain reaction; CXCL5, C-X-C motif chemokine ligand 5; IHC, immunohistochemistry.

To investigate the role of CXCL5 in RSA, we firstly evaluated the expression levels of CXCL5 in placental villous tissues from 15 RSA patients and 13 normal controls by RT-PCR. Lower expression of CXCL5 was found in the RSA group than in the control group (**Figure 1A**). In addition, to further identify immunolocalization and compare CXCL5 levels, human placental villous tissue sections were stained *via* immunohistochemistry (IHC) assay. A weaker stain of CXCL5 was found in villous tissues from RSA patients (**Figures 1B, C**). These data together suggested that CXCL5 expression was downregulated in villous tissues of RSA patients and that lower levels of CXCL5 were positively associated with RSA.

### Expression of CXCR2 in Trophoblast of Human Placental Villous Tissue

**Figure 2:**
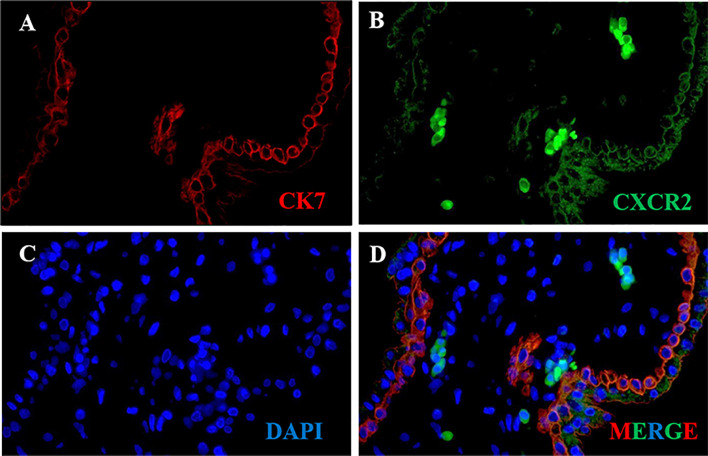
CXCR2 expression in trophoblast of human placental villous tissues. Paraffin-embedded tissue sections were double-stained for immunofluorescence. **(A)** The CK7-labeled trophoblast cells are indicated by red signals. **(B)** The CXCR2 protein is indicated by green signals. **(C)** The DAPI-stained nuclei are indicated by blue signals. **(D)** CXCR2 was expressed in CK7-labeled trophoblast cells and strongly indicated the colocalization of CK7 and CXCR2. CK7, cytokeratin 7; CXCR2, C-X-C motif chemokine receptor 2; DAPI, 4′-6-diamidino-2-phenylindole.

CK7 was recommended as an identification marker for trophoblast (31). To confirm the CXCR2 expression in the trophoblast of human placental villous, we performed colocalization of CXCR2 and CK7 in human placental villous tissue with the immunofluorescence assay. The results confirmed that CXCR2 was expressed in the CK7-labeled trophoblast and suggested that CXCL5 can bind to the trophoblast cells to exert its functions (**Figures 2A–D**).

### CXCL5 Promotes Trophoblast Migration and Invasion *via* Inducing the Epithelial-to-Mesenchymal Transition Process

**Figure 3:**
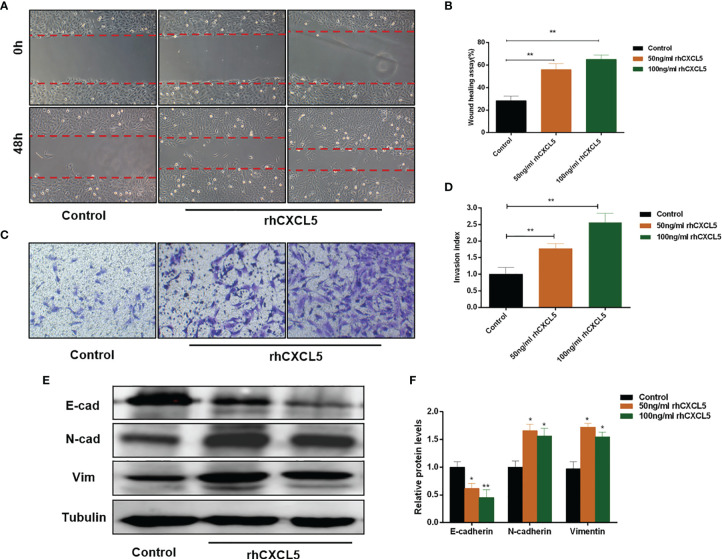
CXCL5 induces the EMT process to promote trophoblast migration and invasion. HTR-8 cells were treated with 0, 50, and 100 ng/ml of rhCXCL5 for 48 h. **(A, B)** Scratch wound healing assay and quantitation were performed to examine trophoblast migration ability. **(C, D)** Matrigel invasion assay and quantitation were used to detect trophoblast invasiveness ability. **(E, F)** Western blotting and quantitation were determined to examine the expression levels of E-cadherin, N-cadherin, and vimentin. Representative images of migratory and invasive cells are presented. Data are presented as the mean ± SD. Results are reported as fold change compared with the control group (*p < 0.05, **p < 0.01). rhCXCL5, recombinant CXCL5; EMT, epithelial-to-mesenchymal transition; E-cad, E-cadherin; N-cad, N-cadherin; Vim, vimentin.

To investigate the effects of CXCL5 on the migration and invasion of trophoblast, we conducted Transwell and scratch wound healing experiments using HTR-8 cells. The results showed that rhCXCL5-stimulated trophoblast cells performed quicker migration than did the control group (**Figures 3A, B**). Similarly, CXCL5-treated groups also showed an increased cell invasion potential than did the control group (**Figures 3C, D**). These results demonstrated that CXCL5 can significantly enhance trophoblast migration and invasion *in vitro*. EMT was regarded as an important process *via* which trophoblast acquired invasive ability (32). To gain insight into whether CXCL5 promoted trophoblast migration and invasion *via* inducing the EMT process, we detected the expression of EMT markers. Western blotting results revealed that CXCL5 treatment significantly decreased the expression of the epithelial marker E-cadherin and increased the expression of the mesenchymal markers N-cadherin and vimentin in HTR-8 cells (**Figures 3E, F**). Based on these findings, CXCL5 can promote trophoblast migration and invasion *via* inducing the EMT process.

### CXCL5 Activates PI3K/AKT/ERK1/2 Pathway to Induce the Epithelial-to-Mesenchymal Transition Process

**Figure 4:**
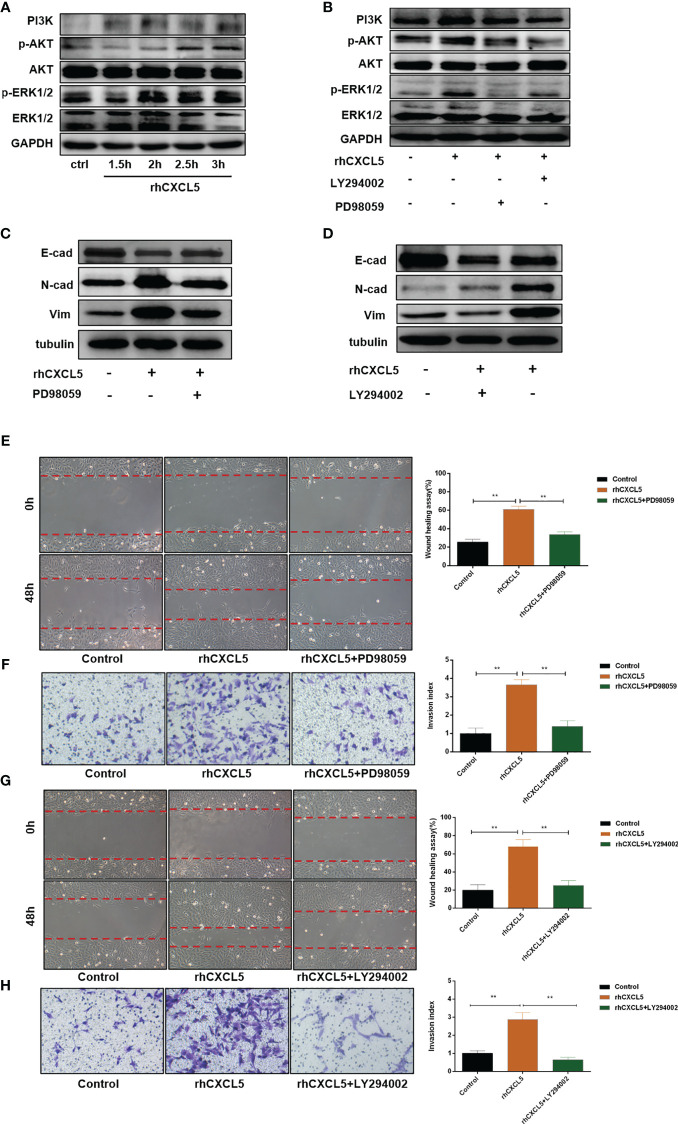
CXCL5 activates PI3K/AKT/ERK1/2 signaling pathway to induce the EMT process of trophoblast cells. HTR-8 cells were pretreated with LY294002 (a PI3K/AKT inhibitor) or PD98059 (an ERK1/2 inhibitor) before treatment with rhCXCL5. **(A)** Western blotting analysis showed the activation of PI3K/AKT and ERK1/2 pathways in HTR-8 cells stimulated with rhCXCL5 for 1.5, 2, 2.5, and 3 h. **(B)** Western blotting analysis presented a notable blocking effect of LY294002 and PD98059 on p-AKT or p-ERK1/2, respectively. **(C, D)** Western blotting was performed to examine the expression of E-cadherin, N-cadherin, and vimentin in HTR-8 cells stimulated with PD98059 or LY294002 for 48 h. **(E, F)** Scratch wound healing and Matrigel invasion assays were performed to detect trophoblast migration and invasion abilities after stimulation with PD98059 for 48 h. **(G, H)** Scratch wound healing and Matrigel invasion assays were used to examine trophoblast migration and invasion abilities after stimulation with LY294002 for 48 h. Data are expressed as the mean ± SD. Column charts were used for quantifications of the migration assay and invasion assays. Results are reported as fold change compared with the control group (**p < 0.01). CXCL5, C-X-C motif chemokine ligand 5.

**Figure 5:**
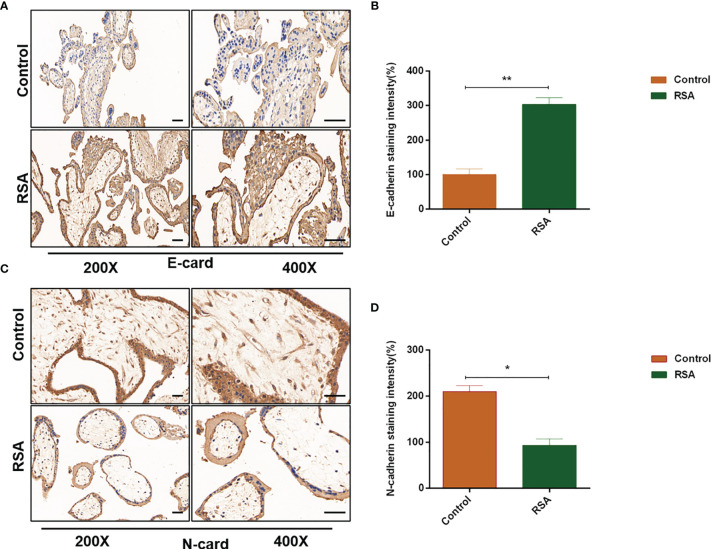
Expression of E-cadherin and N-cadherin in human placental villous tissues from the RSA group and the control group. Placental villous tissues from women with RSA (n = 15) and normal controls (n = 13) were used for the IHC assay. **(A, B)** Representative immunohistochemical staining images and quantification of E-cadherin in the RSA group (n = 15) and normal group (n = 13). **(C, D)** Representative immunohistochemical staining images and quantification of N-cadherin in the RSA group (n = 15) and normal group (n = 13). The staining intensity is quantified using ImageJ. Magnification, ×200 and ×400. Scale bars = 100 μm. Graphs show the mean ± SD. Results are reported as fold change compared with the control group (*p < 0.05, **p < 0.01). RSA, recurrent spontaneous abortion; IHC, immunohistochemistry.

Studies reported that CXCL5 induced the EMT process in cancer cells by activating ERK/Elk-1/Snail, AKT/GSK3β/β-catenin, or ERK/Snail signaling pathways (33, 34). To explore the potential signaling pathway that CXCL5 induced EMT in trophoblast cells, we detected the activity of these pathways. Western blotting analysis showed that the levels of phosphorylation AKT remarkably increased in CXCL5-treated HTR-8 cells, while the total AKT levels did not change (**Figure 4A**). Consistently, PI3K levels also increased. In addition, we also observed apparent activation of p-ERK1/2 (**Figure 4A**). Next, we selected inhibitors specific to ERK (PD98059) and PI3K/AKT (LY294002) pathways for further exploration. A notable blocking effect of LY294002 and PD98059 was observed on p-AKT and p-ERK1/2 levels, respectively (**Figure 4B**). Remarkably, p-ERK1/2 levels also decreased when the PI3K/AKT was inhibited with LY294002, which suggested that ERK1/2 may act as a direct downstream effector of PI3K/AKT signaling (**Figure 4B**). Next, inhibitor pretreatment was initiated 2 h before CXCL5 treatment in HTR-8 cells. The results showed that PD98059 pretreatment significantly reduced the expression of N-cadherin and vimentin but increased the expression of E-cadherin compared with CXCL5 treated alone in trophoblast cells (**Figure 4C**), which indicated that PD98059 reversed the CXCL5-induce EMT process. In addition, wound healing assay and Matrigel invasion assay results also showed that the invasive and migratory activities of cells were reduced when the ERK1/2 pathway was inhibited (**Figures 4E, F**). Similar results were observed in the LY294002 treatment group (**Figures 4D, G–H**). Taken together, these results confirmed that CXCL5 activated PI3K/AKT/ERK1/2 pathway to induce the EMT process of trophoblast cells. We also checked the E-cadherin and N-cadherin expression in human placental villous tissue specimens with IHC assay. The results showed that E-cadherin expression was upregulated (**Figures 5A, B**) and N-cadherin expression was downregulated (**Figures 5C, D**) in placental villous tissues from RSA patients compared with the controls.

### Estrogen, Progesterone, Human Chorionic Gonadotropin, and Decidual Stromal Cells Regulate CXCL5/CXCR2 Expression of Trophoblast

**Figure 6:**
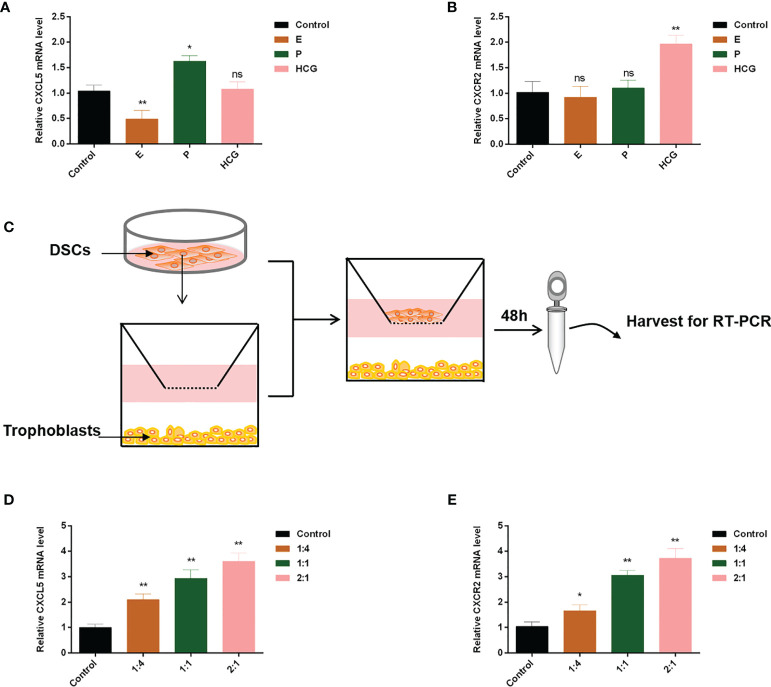
E, P, HCG, and DSCs regulate CXCL5/CXCR2 expression of trophoblast cells. HTR-8 cells were treated with E, P, and HCG at the indicated concentrations for 48 h. **(A, B)** RT-PCR was used to detect the expression levels of CXCL5 and CXCR2. **(C)** A co-culture system of HTR-8 cells and DSCs was taken for 48 h. **(D, E)** RT-PCR was used to investigate the impact of DSCs on CXCL5 and CXCR2 expression of trophoblast cells. Graphs show the mean ± SD. Results are reported as fold change compared with the control group (*p < 0.05, **p < 0.01, ns, no significant difference, *p* > 0.05). E, estrogen; P, progesterone; HCG, human chorionic gonadotropin; DSCs, decidual stromal cells; RT-PCR, quantitative real-time PCR.

Extensive evidence revealed that reproductive hormones and DSCs directly or indirectly affected chemokines expression (35–37). Therefore, to confirm their impacts on CXCL5 and CXCR2 expression of trophoblast cells, we cultured HTR-8 cells in the presence or absence of E, P, or HCG. The results are displayed in **Figures 6A**, **B**. We found that HCG increased CXCR2 expression (*p* < 0.01) but did not affect CXCL5 expression in HTR-8 cells (*p* > 0.05). E was observed to downregulate CXCL5 levels (*p* < 0.01) but did not affect CXCR2 expression (*p* > 0.05). P did not affect CXCR2 (*p* > 0.05) but increased CXCL5 mRNA expression (*p* < 0.05). To assess the effect of DSCs on CXCL5 and CXCR2 of trophoblast cells, we used a co-culture model of different ratios of HTR-8 cells and DSCs, as shown in **Figure 6C**. The results indicated that DSCs can promote the expression of CXCL5 and CXCR2 of trophoblast cells, even when cultured at a lower ratio (DSCs: HTR-8 cells, 1:4; **Figures 6D, E**).

## Discussion

RSA is the most common pregnancy-related complication affecting reproductive-age women. Accumulating evidence has demonstrated that trophoblast invasion is closely associated with embryo implantation and placentation and thus plays an important role in the establishment and maintenance of pregnancy. Moreover, studies reported that insufficient invasion of trophoblast cells can lead to the occurrence of RSA (10, 12). Our previous work also showed that miR-27a-3p/USP25 axis participated in the pathogenesis of RSA through inhibiting trophoblast migration and invasion (11). In the current study, we found that CXCL5 levels were downregulated in villous tissues from RSA patients. Furthermore, we demonstrated that CXCL5 induced the EMT process to promote trophoblast invasion and migration through PI3K/AKT/ERK1/2 signaling pathway. These results together demonstrate that the downregulation of CXCL5 in villous tissue plays an essential role in the pathogenesis of RSA.

The maternal–fetal interface exists an abundant chemokines network, which exhibits main functions in inflammation, immune tolerance, and trophoblast invasion during early human pregnancy (14). Substantial studies have reported the relationship between chemokines and trophoblast invasion. Zhang et al. revealed that CXCL6 restricted human trophoblast migration and invasion *in vitro* (38). Wang et al. pointed out that both exogenous and endogenous CXCL3 regulated trophoblast cells invasion (27, 39). Similar results have been identified in CCL24, CXCL16, CXCL14, CCL14, and CCL17 (37, 40–43). Chemokines have been widely reported in the field of cancer and are associated with angiogenesis, invasion, and metastatic potential of tumors. For instance, Mao et al. found that CXCL5 can enhance gastric cancer cells migration and invasion ability *via* inducing the EMT process (34). Kodama et al. reported that the CCL3/CCR5 axis contributed to esophageal squamous cell migration and invasion (44). Trophoblast cells have much in common with tumor cells (24). Therefore, our current study investigated the effect of CXCL5 on trophoblast invasion. As described above, we firstly detected CXCL5 expression in villous samples from RSA patients and control patients and found a significant downregulation of CXCL5 in the former. Next, we confirmed that human villous trophoblast expressed CXCR2, which was consistent with a previous study, and suggested CXCL5 can exert impacts on trophoblast cells *via* the receptor-ligand binding mechanism (25). We further conducted *in vitro* experiments using HTR-8 cells. Exploring trophoblast invasion relies on a suitable trophoblast line because obtaining pure, primary, first-trimester human trophoblast remains a challenge. Compared with BeWo, JEG-3, and JAR, which are highly malignant and have a substantially different transcriptomic profile from EVTs, the HTR-8/SVeo cell line is reported to contain a heterogeneous population of trophoblasts and has been widely used to investigate EVT biology and functions (45, 46). Our results showed that CXCL5 can promote trophoblast cells migration and invasion. Therefore, the downregulation of CXCL5 in villous tissues of RSA patients leads to inadequate trophoblast invasion and the development of RSA.

EMT is firstly described by Elizabeth Hay and is referred to as a multistep dynamic cellular phenomenon in which epithelial cells lose their cell–cell adhesions and gain migratory and invasive traits that are typical of mesenchymal cells (47). This process is characterized by loss of the membranous epithelial marker E-cadherin, increase of mesenchymal markers including vimentin and N-cadherin, and enhanced migratory and invasive behaviors. It has been reported that EMT participates in embryonic development, tissue repair, and cancer metastasis (48–50). We provided the first evidence that CXCL5 induced the EMT process to enhance trophoblast invasion and migration. Additionally, we also observed the reversal of EMT in CXCL5-reduced villous tissues from RSA patients. These data emphasized the importance of EMT in pregnancy. Interestingly, recent studies have also found that EMT has an intimate association with aerobic glycolysis (51, 52). In addition, Ma et al. have reported that lactic acid, which is a critical metabolite product of aerobic glycolysis, plays a role in trophoblast invasion and angiogenesis (53). However, whether this effect is mediated by EMT induction to link pregnancy requires further investigation.

PI3K/AKT and ERK pathways are reported to play significant roles in the CXCL5-induced cell invasion and the EMT process. Qiu et al. found that CXCL5/CXCR2 axis contributed to the EMT of nasopharyngeal carcinoma cells by ERK/GSK-3β/snail signaling (23). Zhao et al. reported that tumor-derived CXCL5 promoted human colorectal cancer metastasis through the activation of ERK/Elk-1/Snail and AKT/GSK3β/β-catenin pathways (33). In our present study, CXCL5 can activate PI3K/AKT/ERK1/2 pathway to induce the EMT process and enhance trophoblast invasion. These data again highlight the importance of PI3K/AKT and ERK pathway. In addition, PI3K/AKT signaling is also identified as a potential therapeutic target. Epidemiological studies and meta-analyses have shown that the use of statins is closely associated with a reduced incidence of colorectal cancer (54–56). Recently, a new study has revealed that statins can target inhibition PI3K/AKT/mTOR signaling and thus acts on colorectal cancer progression (57). Remarkably, another research has found that pravastatin can successfully prevent fetal death in a pregnant woman with a history of four consecutive pregnancy losses (58). However, whether this effect depends on PI3K/AKT pathway needs more exploration.

It is reported that reproductive hormones E, P, and HCG play important roles during pregnancy and can affect the expression of chemokines within the maternal–fetal microenvironment (36). Currently, we found that P significantly increased CXCL5 expression while E had an opposite function. Our results also showed that HCG upregulated CXCR2 expression, although it did not affect CXCL5 expression. These findings indicated that CXCL5/CXCR2 axis is regulated by local hormones. There is also a close dialogue between trophoblast cells and maternal DSCs at the maternal–fetal interface (59). Co-culture systems comprising decidual fragments and trophoblasts have been widely used to explore their relationship. Li et al. reported that DSCs promoted CCR3 levels of trophoblast cells (37). Our current result confirmed that DSCs significantly promoted CXCL5 and CXCR2 expression of trophoblast cells. However, whether DSCs can induce trophoblast cells to secrete CXCL5 and participate in the regulation of trophoblast invasion deserve further research in future work.

**Figure 7:**
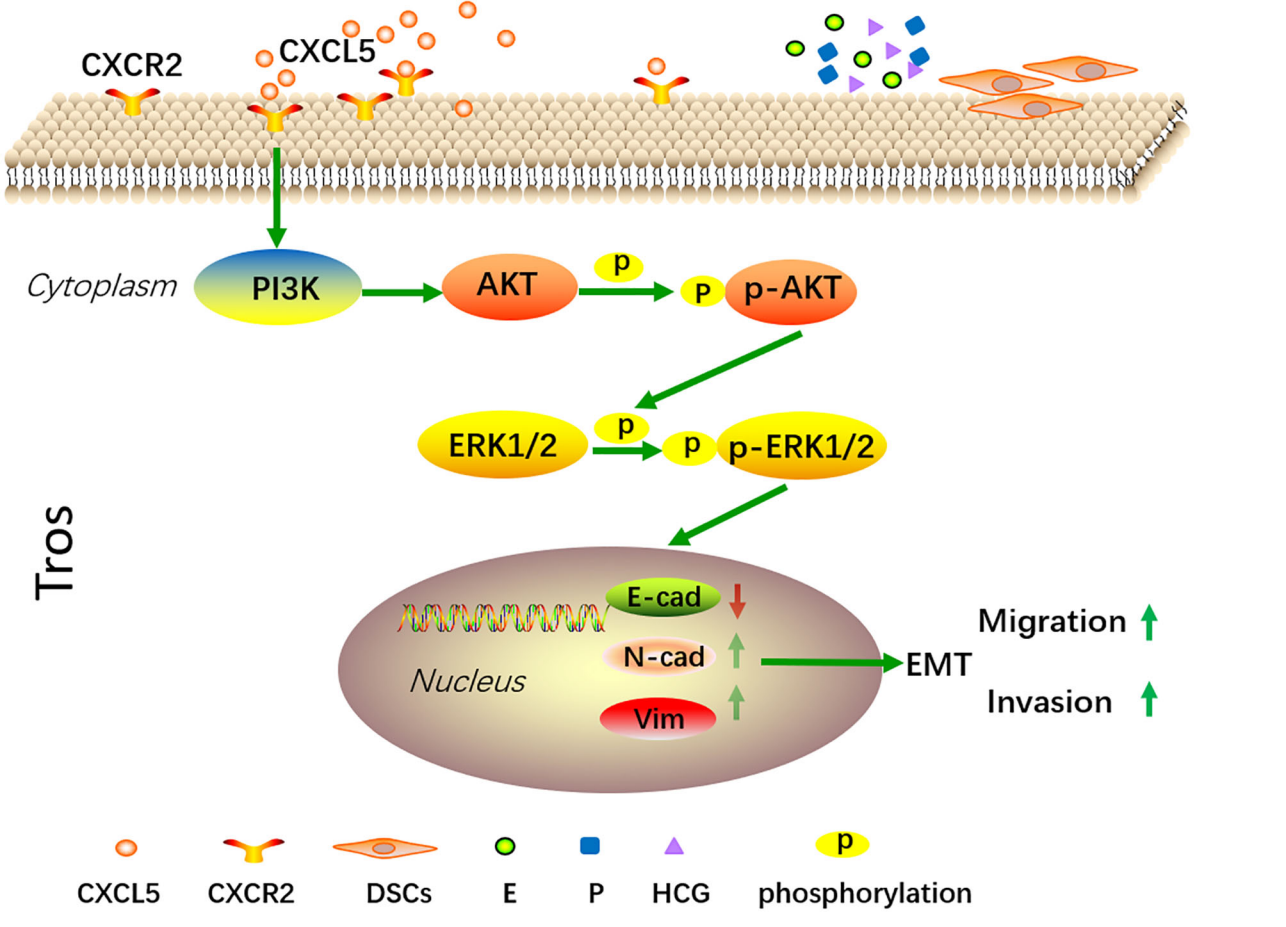
A schematic diagram of CXCL5 promoting trophoblast migration, invasion, and EMT process through PI3K/AKT/ERK1/2 signaling pathway. Trophoblast cells express CXCR2, which is the receptor of CXCL5. Exogenous CXCL5 combined with CXCR2 promotes the EMT process through the activation of the PI3K/AKT/ERK1/2 signaling pathway and consequently increases the invasiveness and migration of trophoblast cells. Conversely, downregulation of CXCL5 results in insufficient invasion and therefore is correlated with the pathology of RSA. In addition, E, P, HCG, and DSCs participate in regulating CXCL5 and CXCR2 expression of trophoblast cells within the microenvironment of pregnancy at the maternal–fetal interface. CXCL5, C-X-C motif chemokine ligand 5; EMT, epithelial-to-mesenchymal transition.

In summary, our present data confirm that CXCL5 levels are significantly lower in human villous tissue from RSA patients. We also demonstrate that CXCL5 can promote trophoblast invasion, migration, and EMT process through PI3K/AKT/ERK1/2 pathway. Taken together, these data indicate that CXCL5 downregulation in villous tissue is correlated with RSA. This provides us with more insights into the molecular pathogenesis of RSA. In addition, we also found that E, P, HCG, and DSCs regulate the expression of CXCL5/CXCR2 in a cell manner. These findings illustrate a new dialogue among chemokines, trophoblast cells, and reproductive hormones in the microenvironment of the maternal–fetal interface (**Figure 7**).

## Data Availability Statement

The datasets presented in this study can be found in online repositories. The names of the repository/repositories and accession number(s) can be found in the article/supplementary material.

## Ethics Statement

The studies involving human participants were reviewed and approved by the ethics committee of Renmin Hospital of Wuhan University. The patients/participants provided their written informed consent to participate in this study. Written informed consent was obtained from the individual(s) for the publication of any potentially identifiable images or data included in this article.

## Author Contributions

TY and YZ supported the research. SZ and JD designed the experiments. SZ performed the experiments and drafted the first version of the manuscript. JW collected the clinical samples and patients’ data. TY, YZ, JD, and JY supervised and revised the manuscript. All authors contributed to the article and approved the submitted version.

## Funding

This work was supported by the following grants: National Key Research and Development Program of China (Nos. 2018YFC1004601, 2018YFC1002804), the National Natural Science Foundation of China (Nos. 81801540, 81771662, 82101749), and the Fundamental Research Funds for the Central Universities (2042021kf0082).

## Conflict of Interest

The authors declare that the research was conducted in the absence of any commercial or financial relationships that could be construed as a potential conflict of interest.

## Publisher’s Note

All claims expressed in this article are solely those of the authors and do not necessarily represent those of their affiliated organizations, or those of the publisher, the editors and the reviewers. Any product that may be evaluated in this article, or claim that may be made by its manufacturer, is not guaranteed or endorsed by the publisher.

## Abbreviations

RSA, recurrent spontaneous abortion; EVTs, extravillous trophoblasts; EMT, epithelial-to-mesenchymal transition; ENA-78, epithelial neutrophil-activating peptide 78; CXCL5, C-X-C motif chemokine ligand 5; CXCR2, chemokine receptor 2; DSCs, decidual stromal cells; E, estrogen; P, progesterone; HCG, human chorionic gonadotropin; PBS, phosphate-buffered saline; RT-PCR, quantitative real-time PCR; IHC, immunohistochemistry; FBS, fetal bovine serum.
